# Ensuring access to essential health products: Lessons from Colombia’s leishmaniasis control and elimination initiative

**DOI:** 10.1371/journal.pntd.0011752

**Published:** 2023-12-01

**Authors:** Carol Vlassoff, Nora Giron, Mauricio Javier Vera Soto, Ana Nilce Silveira Maia-Elkhoury, Arush Lal, Luis Gerardo Castellanos, Gisele Almeida, Christopher Lim

**Affiliations:** 1 School of Epidemiology and Public Health, University of Ottawa, Ottawa, Canada; 2 Strategic Fund, Pan American Health Organization, Washington, DC, United States of America; 3 Subdireccion de enfermedades transmisibles, Ministerio de Salud y Protección Social, Bogotá, Colombia; 4 Neglected, Tropical and Vector-Borne Diseases Unit, Pan American Health Organization, Rio de Janeiro, Brazil; 5 Neglected, Tropical and Vector-Borne Diseases Unit, Pan American Health Organization, Washington, DC, United States of America; 6 Department of Health Systems and Services, Pan American Health Organization, Washington, DC, United States of America; Federal University of Ceará, Fortaleza, Brazil, BRAZIL

## Abstract

**Background:**

This paper identifies opportunities and challenges for leishmaniasis control and elimination in Colombia, emphasizing the role of pooled procurement of essential medicines and supplies. Colombia is among the countries most affected by leishmaniasis globally, and also faces the dual challenge of procuring critically needed medicines in the context of limited national resources. It recently renewed its commitment to the control and elimination of leishmaniasis under its 2022–2031 Public Health Plan (PDSP) through a comprehensive public health approach.

**Methodology/Principal findings:**

The methodology comprises a comprehensive literature review and key informant interviews with leishmaniasis experts from the Colombian national control program and PAHO/WHO, focusing on cutaneous, mucocutaneous, and visceral leishmaniasis. Leishmaniasis is endemic throughout Colombia, with over 11 million people at risk, many of whom live in poverty-stricken, remote and isolated rural areas with limited access to health services. Leishmaniasis care, including medicines, is provided free of charge, but many barriers were nonetheless identified at environmental, population, and health system levels, including the supply of quality-assured medicines. Opportunities to alleviate these barriers were identified, including the support of the PAHO Strategic Fund. Within the context of the sustainable development goals and international leishmaniasis control and elimination targets, Colombian officials have established their own priorities, the highest of which is the reduction of deaths from visceral leishmaniasis.

**Conclusions/Significance:**

The elimination of leishmaniasis as a public health problem presents significant challenges, given its biological complexity and diversity, physical and clinical manifestations, social and economic impacts, frequently burdensome treatment regimens, and insufficient supply of necessary medicines. However, rigorous prevention and control efforts through strong political commitment and a highly motivated workforce can dramatically reduce its burden. Colombia’s new PDSP, which highlights leishmaniasis control, is an opportunity for a revitalized health system response through committed leadership, intersectoral actions, and partnerships with international organizations that share a common vision.

## Author summary

Leishmaniasis is a vector-borne disease transmitted by female sand flies to humans and animals, causing infections ranging from asymptomatic and self-healing cutaneous leishmaniasis to visceral leishmaniasis which is highly lethal if untreated. Although all forms of leishmaniasis are treatable and curable with early diagnosis and treatment, the disease presents many challenges for affected populations, many of whom live in remote rural areas of the developing world, with limited access to public health facilities. Health services also face major constraints in supplying critically needed, yet costly medicines, in the context of limited financial resources. This paper presents the case of Colombia which is among the countries most affected by leishmaniasis globally, and discusses its many challenges in responding to the disease, including the guaranteed supply of necessary medicines. Our results uncover new opportunities for a revitalized health system response through committed leadership, intersectoral actions, such as a One Health approach, and partnerships with like-minded international organizations. These include Colombia’s new 10-Year Public Health Plan that contains specific time-bound goals for leishmaniasis control and elimination as a public health problem, prioritizing a reduction in deaths from visceral leishmaniasis, and potential cost-savings from the acquisition of essential medicines and supplies through the Strategic Fund of the Pan American Health Organization.

## Introduction

Neglected tropical diseases (NTDs) are debilitating diseases that affect over one billion people worldwide [1]. They cause significant human suffering, particularly among poor and vulnerable communities in tropical and subtropical regions, and often lead to devastating personal, social, and economic consequences. Decades-long efforts to alleviate the impact of NTDs have culminated in ambitious global targets for their accelerated control, elimination as a public health problem, and in some cases, complete eradication. “Control” refers to the reduction of disease incidence, prevalence, morbidity, and/or mortality to a locally acceptable level as a result of deliberate efforts; “e*limination”* (interruption) of transmission) refers to reduction to zero of the incidence of infection caused by a specific pathogen in a defined geographical area, with minimal risk of reintroduction, as a result of deliberate efforts; “e*limination as a public health problem”* refers to the achievement of measurable global targets set by WHO in relation to a specific disease; and “e*radication”* refers to the permanent reduction to zero of a specific pathogen, as a result of deliberate efforts, with no more risk of reintroduction. “*Extinction”* is the eradication of the specific pathogen so that it no longer exists in nature or the laboratory, which may occur with or without deliberate efforts. Goal 3 of the United Nations Sustainable Development Goals (SDGs)—ensuring healthy lives and well-being for all—includes the target of ending the 20 NTD epidemics by 2030, thereby making the attainment of the SDGs conditional upon achieving this NTD target [1]. Leishmaniasis is among the NTDs included in the target.

The WHO Roadmap for Neglected Tropical Diseases 2021–2030 [1] builds upon this target, as well as the previous 2012 NTD Roadmap [2], with internationally-agreed global targets for the eradication, elimination, or control of major NTDs. Importantly, the new Roadmap recognizes the need for “radical change … to integrate and mainstream approaches into national health systems and coordinate action across sectors… to improve the cost–effectiveness, coverage and geographical reach of programmes” [1,p. viii). The emphasis on NTDs as integral components of national health systems represents a recognition that the fight against NTDs must be country-led, with clear roles for stakeholders, taking into account the diverse cultural contexts of affected populations [1]. This includes, among others, strengthening the capacity of national health systems to provide high-quality services through existing infrastructure, including equitable access to comprehensive treatment and care and promoting a systemic approach through support and coordination with other sectors, including vector control, zoonosis and environmental programs, housing, water, and sanitation. In this paper, we focus on the opportunities and challenges of implementing this integrated approach to address NTDs in the region of the Americas, with particular emphasis on the role of pooled procurement of essential medicines and strategic public health supplies. We have chosen the example of the control and elimination of leishmaniasis in Colombia and the role of the Pan American Health Organization’s (PAHO’s) Strategic Fund to illustrate this.

Leishmaniasis is a vector-borne disease transmitted by female phlebotomine sand flies to humans, presenting zoonotic and anthroponotic cycles. In the Americas, their complex biological cycle is only zoonotic, in which vector transmission is to animals and humans as hosts. It has an array of clinical forms, from asymptomatic infections and less severe self-healing cutaneous leishmaniasis (CL) to mucocutaneous (MCL) and visceral leishmaniasis [VL), the latter of which is highly lethal if left untreated [3,4,5]. Most leishmaniasis deaths are associated with visceral disease, progressing through fevers, malaise, loss of appetite and weight loss associated with anemia, splenomegaly, and hepatomegaly [6]. Hemorrhages and bacterial infections are important and frequent risk factors for death in patients with VL. Other risk factors for death include jaundice, thrombocytopenia, coinfection with human immunodeficiency virus (HIV), diarrhea, older age, neutropenia, and dyspnea [7]. Importantly, all forms of leishmaniasis are treatable and curable with early diagnosis and prompt and effective treatment to prevent further disabilities and death [7].

We present the specific case of leishmaniasis in Colombia because it is among the most affected countries globally and ranks second in the Americas, after Brazil [8]. Colombia also confronts the dual challenge of procuring quality-assured and cost-effective medicines and public health commodities for affected populations in the context of limited national resources. Notably, Colombia recently adopted a concerted approach to the control and elimination of leishmaniasis as a public health problem under the umbrella of its new 10-year Public Health Plan (*Plan Decenal de Salud Publica 2022–2031*) [9], discussed in more detail below, and is in the process of strengthening its leishmaniasis control efforts through a comprehensive public health approach. This offers a unique opportunity to evaluate important, real-time insights that may be useful for other countries facing similar challenges, as well as NTD elimination efforts more broadly.

## Methods

This paper is based on a comprehensive review of relevant peer-reviewed and grey literature, complemented by key stakeholder interviews with Colombian NTD and leishmaniasis experts from the Ministry of Health and Social Protection and PAHO/WHO regional, sub-regional, and country-based experts. The literature review comprised a search of databases in English and Spanish, using the University of Ottawa Library’s search tool (OMNI), which searches the University of Ottawa’s collection and other databases, including PubMed (Medline), SCOPUS, Web of Science, Medline (OVID), and the University’s Research Institutional Repository. Google databases and SciELO were also searched for relevant scientific articles, reports, global accords and declarations, and national plans and policies, with a focus on challenges and opportunities in leishmaniasis control in Colombia, regionally (Latin America and the Caribbean), and globally. Keywords searched included: “cutaneous/visceral leishmaniasis and medicines/public health supplies”; “procurement and leishmaniasis medicines/public health supplies”; “benefits/advantages of pooled procurement and leishmaniasis medicines/public health supplies”; “Strategic Fund (PAHO) benefits and leishmaniasis medicines/public health supplies”; “Strategic Fund (PAHO) and access to medicines”; and “pooled procurement and access to medicines”. More details on the literature search are included in S1 Table.

Our stakeholder interviews were conducted virtually (using Zoom and Teams). They included interviews with PAHO/WHO staff at regional, sub-regional, and country levels, as well as in-depth interviews with Colombian public health officials. The interviews with PAHO’s regional and sub-regional staff discussed the response of the Strategic Fund regarding the range of leishmaniasis medicines and other public health products offered to the countries of the Americas, with a focus on Colombia, and challenges and opportunities for greater impact. The multi-country staff interview concerned country experiences in the use of the Strategic Fund’s medicines and supplies, especially its constraints and future opportunities in this regard. The latter used open-ended questionnaires prepared by the principal author and revised by PAHO/WHO regional and sub-regional experts. It was held in Spanish and lasted approximately 1.5 hours. Three in-depth interviews, also using open-ended questions, were conducted with experts in Colombia, including country-level PAHO/WHO and national-level Colombian experts. The latter involved senior public health officials responsible for vector-borne diseases in the Ministry of Health and Social Protection, including leishmaniasis, and for the management of medicines and public health supplies. The interviews were conducted in Spanish and transcribed into English for discussion and analysis by the co-authors at several opportunities during the preparation of the paper.

Our findings reflect the most recent information available at the time of the government transition in Colombia, June 2022, just after the release of the latest 10-year Public Health Plan. While subject to change, it is expected that the overall NTD and leishmaniasis goals and strategies will remain essentially the same, and these will be continually re-evaluated by Ministry officials over the next ten years in terms of achievements, including their conformity with global elimination goals.

## Results

### Leishmaniasis in Latin America and Colombia: A brief history

#### Epidemiological characteristics

In the Americas, recent regional and country-level data from 17 countries reported an annual average of 46,684 cases of CL and MCL, and 13 VL endemic countries reported an average of 3,086 cases per year from 2012–2021. Brazil, Colombia, and Peru together reported 68% of the total cases of CL and ML in the Region [10]. Environmental, social, and economic factors play a role in differences between countries [11,12], including the transmission of cases within and near a dwelling. Overall, the Americas has experienced a decrease in new cases of leishmaniasis over the last few years due, at least partially, to improved access to timely laboratory diagnosis and treatment. However, cases have remained stable or even increased in some countries [10]. VL cases have declined significantly over the past two decades, but their lethality remains a concern [12]. Leishmaniasis is endemic throughout Colombia in all but three provinces (Bogotá D.C., San Andrés Islands, and Atlántico), with more than 11 million people at risk [13], mainly in rural areas, with a wide geographic distribution of vector species and parasites [14,15,16]. The total number of leishmaniasis cases fluctuated considerably in the past, with an annual average of 5,900 cases in the 1990s, to an average of 17,100 cases in 2005 and 2006, followed by a downward trend to 2021, when there were 6,175 cases recorded [8]. CL accounts for 98% of cases, with 77% in men, and 67.7% of cases in the 15–44 age group [8]. MCL accounts for 1.4% of cases, and VL for 0.2% [13]. While only 183 cases of VL were reported from 2013–2018, its focal areas are found in 47 municipalities, exposing approximately 800,000 people in two large municipalities, Cartagena and Neiva, and in smaller rural population centers [13]. The largest recorded outbreak of CL occurred during 2005–2009, with more than 35,000 cases [17]. Another significant outbreak occurred in the Andean valleys in 2003–2004, with 2,810 CL cases, of which 80% were in the 15–44 age group [17]. According to MSPS officials, since 2018, surveillance efforts have been complicated due to the COVID-19 pandemic, when active case detection was not possible.

#### History of control efforts

The Colombian health system is composed of the following main components: the Ministry of Health and Social Protection (MSPS), territorial health directorates, health-promoting entities (EPS), health-providing institutions (IPS), financing/insurance entities and mechanisms, and other key collaborating institutions. In 2017 the Universal Health Coverage Forum [18] launched the people-centered health services approach, emphasizing community participation in the development of health policies and services, focused on community needs and preferences, with a view to achieving universal health coverage. In this vein, Colombia’s 32 departments and five districts all play a role in leishmaniasis control, which was governed from 1996 by Resolution 4288 which designated that leishmaniasis services be provided free of charge under the Basic Health Plan (*Plan de Atención Básica*) [19]. This was later replaced by Resolutions 518 of 2015 [20]) and 3280 of 2018 [21] that authorized the free provision of leishmaniasis services and capacity building, including health education and communication, and services to prevent and control vector-borne diseases, including leishmaniasis.

Colombia’s leishmaniasis program has historically been governed by a series of national public health plans, including the national 10-year Public Health Plan, 2012–2021 (Plan Decimal de Salud Pública, 2012–2021] (PDSP) [22], under which the Strategic Plan for Leishmaniasis 2018–2022 [13] was developed. The former encompassed strategies for disease prevention, treatment and rehabilitation, as well as a broader intersectoral approach that emphasized the importance of reducing the economic and social burdens placed upon the population by the disease. Colombia’s new10-year Public Health Plan 2022–2031 (PDSP) [9] takes an even stronger intersectoral approach, recognizing the impact of social, economic, and cultural inequalities on the burden of disease, and is founded on guaranteeing the fundamental right to health, wellbeing, and quality of life for the Colombian people. It seeks to act upon the social determinants of health through territorial, institutional, and social strategies, to strengthen public health management across geographical territories and with the involvement of all levels of stakeholders. Its four main goals are to [1] advance toward guaranteeing the fundamental right to health through intersectoral and societal actions to positively affect the determinants of health; [2] advance toward the improvement of living conditions, wellbeing and quality of life by reducing social inequalities in health; [3] reduce avoidable mortality, morbidity, and disability, and their impact on years of life lost and healthy life years; and [4] improve environmental health by protecting ecosystems, mitigating the effects of climate change, and consolidating healthy and sustainable territories. The PDSP, and its emphasis on social solidarity, territorial health sector strengthening, intersectionality, human rights, and the reduction of inequalities, provides an important platform for accelerating action on the challenges posed by multi-dimensional, complex NTDS, including leishmaniais.

As of 2022, Colombia had selected two targets for leishmaniasis control and elimination, one each for CL and VL, which are incorporated into the new PDSP [9]. Its targets have been defined within the context of PAHO/WHO’s regional indicators and adjusted to reflect local realities in the focal leishmaniasis areas. The target for VL in the new PDSP anticipates that the conditions necessary for its elimination as a public health problem will be achieved in 20% of the 47 focal municipalities by 2031 (≤1 case per 10.000 inhabitants), with another 20% on the path toward elimination. Hence 40% of endemic municipalities are targeted for VL elimination, or near elimination, by 2031. Data reported in Colombia’s Strategic Plan for Leishmaniasis 2018–2022 [13] indicate that there was a progressive and sustained reduction in the number of VL deaths by 50% in all departments and territories by 2021. While this is clearly an impressive achievement, it is unlikely that the other regional target of a reduction to ≤1 case per 10,000 inhabitants in leishmaniasis focal areas will be achieved by 2031, according to MSPS authorities, but they also point out that using national-level elimination targets can mask the actual situation in endemic areas and create a false impression that the disease is no longer a priority, thus undermining disease control efforts. For example, if the entire Colombian population was taken as the denominator, a large part of whom live in non-endemic areas (including the capital, Bogota, with over 8 million people), the international target could appear to be reached, while remaining a significant challenge in chronically endemic areas. Furthermore, understanding the process by which targets are achieved (or not), such as which interventions were successfully employed, is key to creating sustainable solutions.

Colombia has established a target of reducing the proportion of accumulated cases of CL from the baseline of a 9.5% reduction in children aged ≤10 between 2012–2019 to a further 10% reduction by 2031. The proportion of cases in children aged ≤10 is used as an indicator of disease prevalence because it is assumed that transmission largely occurs inside dwellings where children tend to spend most of their time. Again, this is below the PAHO/WHO target of reducing by 50% the proportion of children aged ≤10 infected with CL/MCL, due to the many challenges presented by this disease in focal areas (see below) and the inability of the service provider network to reach all necessary populations (MSPS officials).

### Leishmaniasis control and elimination in Colombia: Challenges and opportunities

#### Environmental challenges

Like other NTDs, leishmaniasis affects some of the world’s poorest people and is therefore inherently and inextricably linked with social determinants of health (SDH), including inadequate housing; lack of access to financial resources, water, sanitation, and nutritious food; population movements; environmental degradation; and urbanization [7,23]. In Colombia, many conditions favor the transmission of leishmaniasis, such as the mobilization of large population groups from rural to urban areas where they establish settlements in communes and marginalized communities, often coexisting with domestic animals that attract and foster sand fly vectors from nearby surroundings [24].

Despite the challenges such environments present for leishmaniasis prevention and control, the PDSP offers a constructive framework to address these SDH, including promoting the concept of healthy and sustainable territories, fostering the protection of ecosystems, and mitigating the effects of climate change. Concrete actions include a better articulation of public health management and governance in support of primary health care, intersectoral actions and community participation for collective risk management, and vector surveillance and control measures. The latter include the identification of their presence in the intra- and peri-domicilium area, and if necessary, appropriate application of residual insecticide in homes; insecticide-treated bed nets (although the use of bed nets is problematic in some endemic areas of Colombia (Amazon, Atlantic) where people sleep in hammocks, and the nets are not adapted to this application, according to (MSPS officials); and in areas with VL, if indicated, measures to reduce contact of the vector with canine populations [24].

Importantly, Colombia has an integrated approach to collective interventions to control and eliminate vector-borne diseases, including malaria, arboviruses, leishmaniasis, and Chagas disease, whose outbreaks often occur in the same zone sharing the same environmental risks. These are addressed at the national level through Comprehensive Health Care Roadmaps or RIAS (*Rutas Integrales de Attention en Salud*) for harmonized planning related to health maintenance in different settings (domestic, community, educational, institutional, and informal labor sector). As an illustration of this intensified and intersectoral approach, a pilot project for VL elimination was initiated in Neiva, the capital city of the Department of Huila, coordinated by MSPS and executed by the municipal Secretary of Health with support from PAHO/WHO, which is expected to serve as a demonstration project for other endemic municipalities [25]. Neiva has a high level of poverty [26] with poor sanitation and favorable ecological conditions for leishmaniasis transmission, including a high prevalence in dogs. The control strategy includes strengthening surveillance and the integrated management of vectors, reducing transmission risks through an intersectoral approach, improving diagnosis, treatment, and follow-up of leishmaniasis patients, and community participation, in line with the PDSP. According to MSPS officials, deaths in Neiva have significantly declined as a result of these interventions, but continued efforts to contain leishmaniasis outbreaks will be needed because the parasites and vectors are constantly adapting and spreading into new habitats.

A lingering threat to VL prevention is its high prevalence among domestic canines (20%). To address this problem in Neiva, the strategy of culling positive dogs (in an ethically correct manner) was adopted. With the help of veterinarians and the sensitization of community members, the procedure was generally accepted, but the practice is problematic in the long term. Recent evidence from elsewhere suggests that insecticide-impregnated dog collars are a more effective and durable solution. Studies developed with Impregnated collars with slow-release of deltamethrin 4% have demonstrated a 50% effectiveness in decreasing the prevalence of canine leishmaniasis in the Americas, as well as in the abundance of *Lutzomyia longipalpis*, the most important vector of VL in the Americas, in the intra- and peri-domiciliary areas [27,28]. Colombian authorities are planning to implement this intervention in Neiva because a cost-effectiveness study in Brazil showed that collars impregnated with deltamethrin were considered highly cost-effective in preventing canine VL when used in a public health program [29].

#### Population-related challenges

In Colombia and many other leishmaniasis affected countries across the Americas, people most at risk of infection experience not only socioeconomic, structural, and environmental disadvantages but also susceptibility to diseases of poverty, such as malaria, other parasitic infections, and malnutrition. This is due to their often poor access to health, sanitation, and educational services [30]. Anecdotal evidence from the interviewed health authorities suggests a link between poor nutrition and VL infection in children, as it seems that only children with severe malnutrition become ill, even though a high percentage of children in an endemic area may be infected. Many vulnerable citizens living far from health centers have to travel on foot to seek care [30], and those who are day laborers cannot spare the time and resources to travel or take their children for care. Additionally, they are often referred to a higher level when reaching first-level care, entailing even more time and expense [30]. The long treatment protocols requiring people to be treated for 20 days consecutively for CL, for Pentavalent antimonial (anti-parasitic) injections in the buttocks need to be administered by a specially trained health worker because they can cause serious adverse effects and are painful, creating fear and discomfort among patients who may delay seeking such services until their illnesses become severe or when traditional remedies fail [30].

Many people living with CL and MCL experience significant social stigma and psychological distress as a result of its disfiguring skin lesions, leading to reduced quality of life, self-deprecation, decreased social participation, and fear of infecting others [30,31]. A recent study in two endemic areas in Colombia found significant levels of perceived or anticipated stigma, mental distress, and restricted social participation among patients with CL, and these associations were higher among rural, compared to urban communities [31]. An earlier study in Colombia found that the type and visibility of the lesions, as well as how long a person had lived with it, affected how stigma was experienced [32]. For example, among new migrants to the Darién area where the research was conducted, becoming infected with CL had a positive effect: the endurance of its course and the curing and scarring of the lesions were taken as a sign of acceptance into the community, whereas the local African descendant population found it incapacitating and disfiguring [32]. There is generally less documentation of stigma and psychological distress attached to VL, but some studies elsewhere have shown associations between VL and reduced quality of life and risk of mental health problems [33].

Colombia’s new 2022–2031 Public Health Plan [9] places considerable emphasis on equity, thus providing an opportunity to address inequities in the leishmaniasis response beyond the traditional measures of mortality, morbidity, and incapacity. This may be particularly useful to examine how targeted interventions can reduce the accumulated socioeconomic and environmental disadvantages linked to related diseases of poverty, a goal that most departments and districts are actively working toward [MSPS officials). These efforts are particularly critical for reducing CL outbreaks and preventing transmission within the home, where continuing infections among children under ten years of age remain a concern. MSPS officials indicated that this will require a shift from the current biomedical approach, based on acquiring and delivering medicines, to a prevention-based approach, based on accelerated efforts to educate and involve community members in leishmaniasis control. It will also involve capacity-strengthening to investigate leishmaniasis outbreaks in focal areas, a challenging task due to its multiple species of parasites, vectors, and transmission scenarios with multiple risk factors and determinants that necessitate responses and resources to be tailored to each individual outbreak. Scaling up of VL control will be based on learnings from the Neiva experience, starting first with departments with culturally similar populations (Tolima and Cundinamarca) but with different models of operation (staffing, management, etc.). While a template applicable to the whole country is not possible, they will look for common elements to generate synergistic goals, guidelines, and verification criteria while allowing for regional adaptations.

#### Health system challenges

In Colombia, leishmaniasis diagnostics and treatments are mostly in line with the recommendations for comprehensive patient care endorsed by PAHO/WHO and other leishmaniasis experts [34]. Dealing with the many challenges posed by leishmaniasis in Colombia requires a responsive health system coordinated across various sectors at municipal, territorial, and national levels; however, several limitations have been identified in the effectiveness of this response in meeting the needs of affected communities. These include a reliance on national regulations that do not reflect the realities of the affected communities or have inadequate health infrastructures to implement them. A further constraint is the lack of health personnel trained in administering CL treatment at the first level of care, which results in costly delays for patients who are referred to higher levels of the system. New approaches are needed to improve access to treatment, including health team home visits to remote rural areas [30] and capacity-building programs for community health workers to address their own fears and lack of information concerning leishmaniasis in order to better respond to patient needs (MSPS officials).

Implementing local treatments and training for practitioners at the first level of care, such as the application of intralesional pentavalent antimonials and thermotherapy, may further help address this need [34]. In Colombia, thermotherapy, the application of local heat over the lesion and surrounding areas, is recommended by some as a local remedy for CL. A recent study showed thermotherapy to be a cost-effective strategy compared to the use of Pentavalent antimonial (anti-parasitic) injections, with multiple benefits, including better patient compliance, simplicity of application, safety, and lower costs [35]. Colombia’s recently updated guidelines [36] contain a recommendation for use of thermotherapy for pregnant women, when indicated, which is in line with PAHO/WHO’s 2022 recommendations [34]. This technology is a great opportunity with a higher safety profile but it requires the regulatory authority (INVIMA) to allow its importation, commercialization, and use. Also, to support its wider use, officials consider that further evaluation within endemic areas is needed to assess its cost-effectiveness vis-à-vis existing treatments, including the costs of equipment, training and implementation, and restrictions on its application.

Colombia’s new leishmaniasis guidelines, which contain extensive information on its epidemiology, geographical areas, risk factors, and populations most affected; guidelines for diagnosis and treatment; lines of responsibility, and detailed duties at all levels of the health system [36] clearly demonstrate the complex demands that leishmaniasis entails—from the MSPS level overseeing operations to the local level with medical practitioners and community health workers. It is recommended that diagnosis and treatment of CL be done at the first level of care because diagnosis through direct examination involves minimal cost and has a high sensitivity, at an estimated 85%-90%. This is done through smear diagnosis and biopsy, where available. Offering timely diagnosis to initiate treatment is considered the most important measure in the control of leishmaniasis and the priority activity that the health system should take for the care and control of this disease. The other forms of leishmaniasis must be diagnosed and treated by specialists at higher levels of care [36]. Diagnosis and quality control are overseen by the Department of Public Health Laboratories (Laboratorios de Salud Pública Departamental) which coordinate, at the department level, the training and competency assessment of all persons performing leishmaniasis diagnostics, in coordination with the National Institute of Health, and in accordance with international standards recommended by the WHO.

#### Ensuring access to quality-assured medicines and other public health products

Adequate planning and estimation of annual leishmaniasis drug supply requirements is a further challenge for the health system. Because leishmaniasis products are mostly single-source, it is especially important to avoid stock-outs and plan accordingly. Improving the estimation of needs for medicines and supplies needs to be urgently addressed through capacity-building, availability and use of tools, and better coordination between the national level and territories to improve transparency and efficiency. The new leishmaiasis guidelines mandate that a reliable stock of medicines for leishmaniasis treatment be furnished in a timely manner via the relevant departments and municipalities to the health-providing institutions (IPS) in leishmaniasis-endemic areas [36]. This will require heightened training and efforts in the estimation and forecasting of needs, timely requests to the MSPS, adequate storage and management of inventories, stock rotation and management, and systematic monitoring of stock-outs and conditions of use in the IPS. Consideration of possible under-reporting of cases should also be included in these processes. As with other vector-borne diseases, the Ministry makes annual drug supply projections based on the notification of the territorial entities; hence, the involvement of health professionals throughout the country is of paramount importance in ensuring efficient planning and implementation.

Pharmacovigilance is another important oversight activity, including monitoring of patients during treatment and follow-up for non-adherence and adverse effects, especially those with comorbidities or previous leishmaniasis infection. However, most of the measures recommended to monitor drug toxicity, such as diagnostic tests, and laboratory analyses before and during treatment, are not feasible in endemic rural areas [31]. Further, patients should be monitored for relapses for several months after recovery (CL patients for at least 6 months, ML and VL patients for 12–24 months) but there are currently no mechanisms to encourage patients to report for check-ups. Greater involvement of pharmaceutical manufacturers in pharmacovigilance and a community-based surveillance system would help to address these issues.

The timely supply of medicines and other necessary public health products involves several challenges specific to leishmaniasis. These include registration, quality control, pharmacovigilance issues, and challenges related to the medicines themselves. As noted above, the leishmaniasis guidelines address the issue of pharmacovigilance, aimed to strengthen the surveillance of adverse drug reactions with specific functions identified for different levels of health care providers, and to link patients’ clinical history, identification, and lot number of the drugs used [34]. Nonetheless, the fact that some leishmaniasis medicines are not registered in most countries, including Colombia, poses problems for pharmacovigilance and quality control. Diagnostic tests and leishmaniasis medicines are expensive and complicated to administer, with potentially severe and toxic side effects [31]. Aligning expert advice with local realities is a further challenge. For example, the PAHO/WHO guidelines for the treatment of adult patients with CL strongly recommend miltefosine and intralesional antimonial pentavalent over the currently used pentavalent antimonials because of their reduced toxicity, acceptability, and ease of administration (local and orally as opposed to intravenous or intramuscular injections) [34]. However, with current resources, Colombia could only support 30% of the cases, prioritizing patients according to age and preexisting conditions, thus leaving 70% of patients untreated. Other options, such as amphotericin, are even more expensive, costing six or seven times more than miltefosine. Colombia has a local manufacturer of miltefosine, which could help reduce costs. Still, its production capacity is limited, and local physicians, who are trained in and accustomed to using pentavalent antimonial injections, would need convincing and retraining to adopt the new clinical guidelines (MSPS officials).

**Fig 1:**
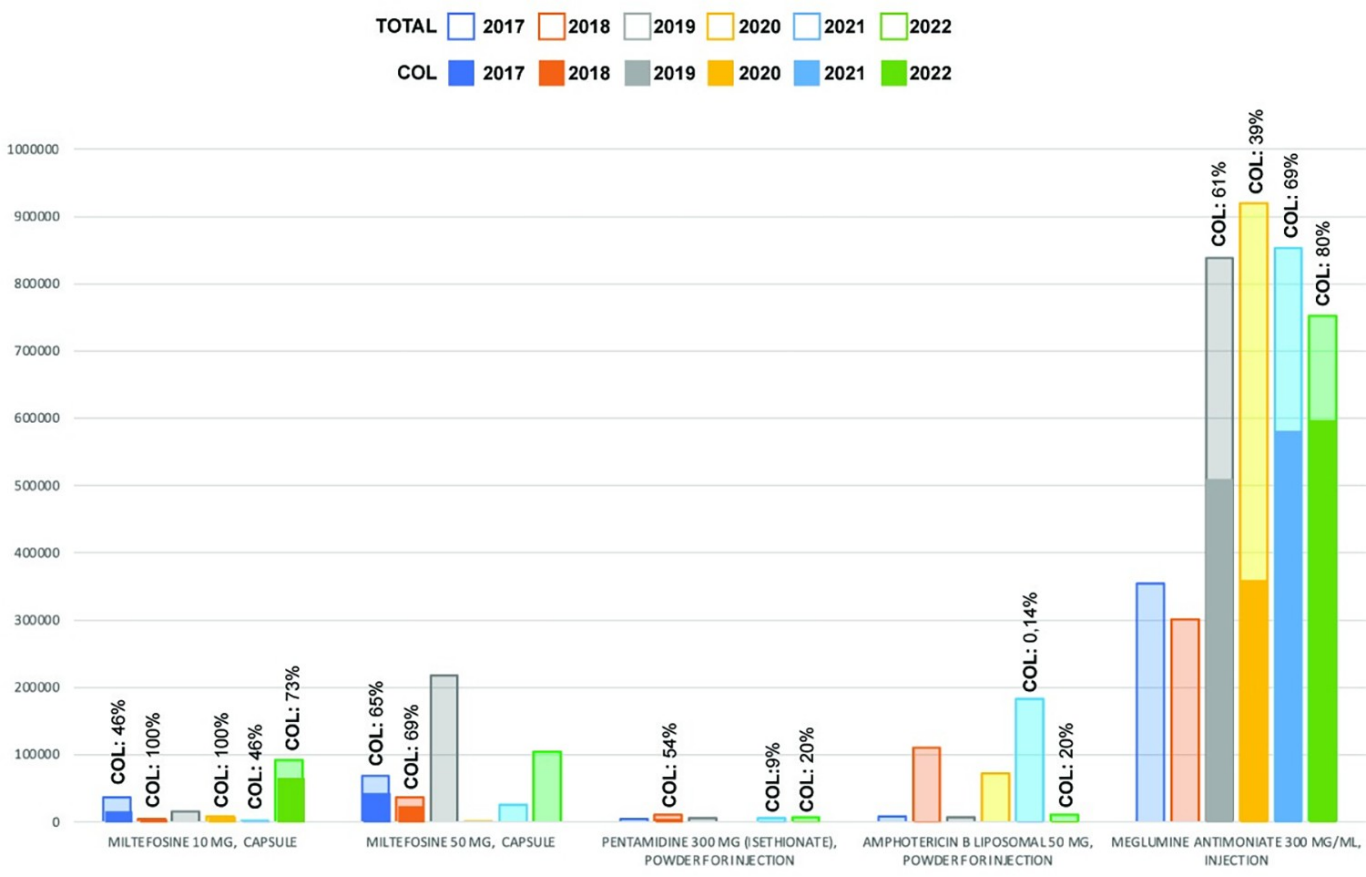
Leishmaniasis medicines supplied by Strategic Fund 2017–2022.

In an effort to facilitate a more equitable procurement process for countries in the Americas, PAHO’s Strategic Fund expanded its list of leishmaniasis available medicines. This list now includes meglumine antimoniate; liposomal amphotericin B, amphotericin B deoxycholate, miltefosine (10 and 50 mg) and pentamidine isethionate. Over the past six years, from 2017 to 2022, the Strategic Fund has supplied essential leishmaniasis treatments to 16 countries, including Colombia, which ranks as the second largest purchaser of leishmaniasis medicines. Of the total purchases made by all countries from the Strategic Fund during this period, Colombia’s procurement of meglumine antimoniate accounted for a significant 51.5% of the total, while its acquisition of miltefosine (10 mg) comprised a substantial 79% (Fig 1).

**Fig 2:**
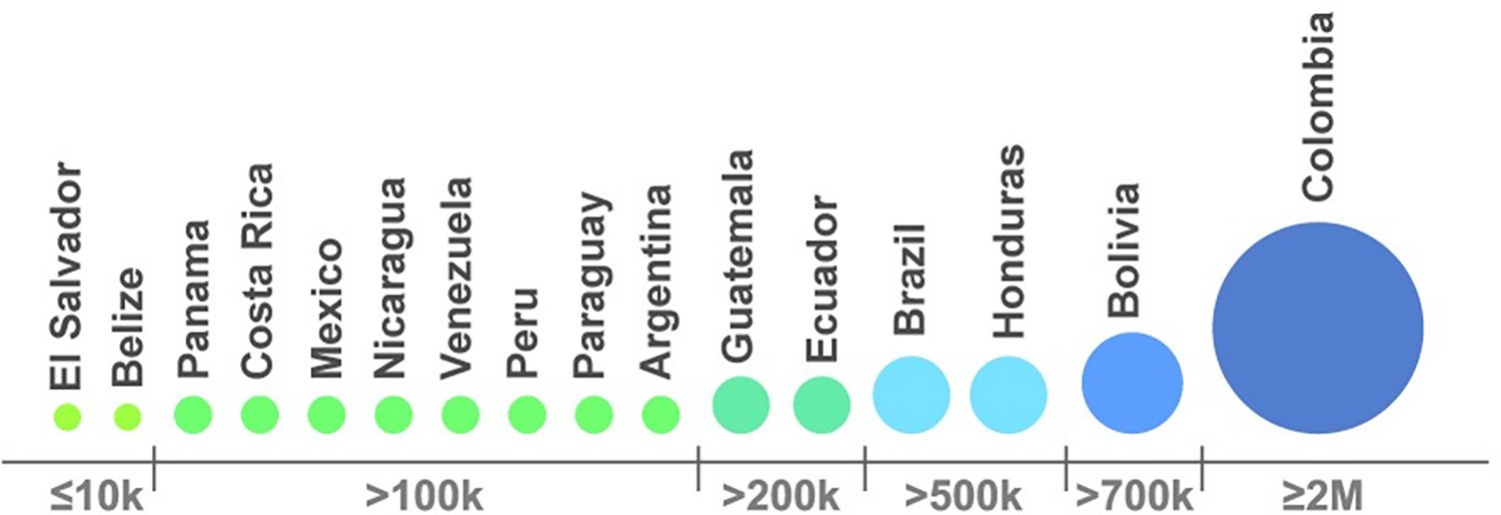
Countries procuring antileishmaniasis medicines through the Strategic Fund, 2017–2022.

The leishmaniasis medicines were procured at competitive prices through negotiations, thereby improving the quality and efficiency of acquisitions and expanding access and coverage for affected populations. This approach not only benefited Colombia but also had a positive impact on the entire region, as all other countries benefited from the single negotiated price (Fig 2). In addition, the Strategic Fund established long-term agreements with exclusive pharmaceutical suppliers for two products, miltefosine and pentamidine, which are difficult to acquire, have low purchase volume, and are generally not available in regional pharmaceutical markets. Since these products are not of competitive interest to the pharmaceutical industry, they are not available in most countries; hence, the only means of acquisition is through the Strategic Fund. Moreover, the Strategic Fund ensures the quality of antileishmanasis medicines through compliance with stringent eligibility criteria and post-market surveillance.

In the context of an ongoing initiative to enhance collaborative planning and ensure timely deliveries to beneficiary countries, the Strategic Fund has implemented a standardized protocol for gathering projected annual requirements for leishmaniasis medicines in order to consolidate demand, leading to a significant and consistent reduction in related product costs. A coordinated presentation of demands from all countries procuring from the Strategic Fund could further attenuate these expenses. However, it must be recognized that, while that meeting the demand for medicine improves treatment and access, the incidence of leishmaniasis is influenced by a multitude of factors, and that an integrated strategy to reduce cases and their spread must be implemented. Such a strategy should include prevention, surveillance and vector control, public health education, and improving living conditions. Adopting a comprehensive approach to these factors is vital for a meaningful decrease in leishmaniasis transmission and prevalence in impacted areas.

Another benefit of the Strategic Fund is the availability of a credit line from its capital account that participating countries are able to use, if necessary, to address the time gap between budgeting and approval of funds, a challenge faced by Colombia. PAHO’s Loans and Donations Platform also facilitates bilateral country-to-country horizontal cooperation through the efficient and equitable lending of needed products [37]. Efforts are continuing to improve pooled purchasing mechanisms, supported by multilateral technical cooperation, to schedule production and secure deliveries in shorter periods.

## Discussion

The control and elimination of leishmaniasis as a public health problem presents significant challenges, given its biological complexity and diversity, physical and clinical manifestations, social and economic impacts, frequently burdensome treatment regimens, and insufficient supply of necessary medicines. Because leishmaniasis affects mainly marginalized and vulnerable populations, often in dispersed and isolated locations, it tends to be neglected by mainstream health care systems, especially in the face of competing health priorities. However, rigorous prevention and control efforts through strong political commitment and the resolve of a highly motivated health workforce can dramatically reduce the burden of leishmaniasis. This has been shown with VL in Nepal, where easy-to-use diagnostic tests, medicines, and efficient vector control methods reduced its incidence to a historic low [38]. For Latin American countries, VL control is more challenging, given the zoonotic cycle, compared to the Nepalese anthroponotic cycle. Its presence in domestic animal reservoirs in Latin America also impedes the interruption of its transmission.

In Colombia, reducing deaths from VL is considered the highest priority for leishmaniasis control, using a step-by-step strategy in priority focal areas that emphasizes improved case management through strengthened service provision networks at the first level of care, including rapid diagnostic tests, effective disease surveillance, targeted interventions, and integrated vector control. Enhanced partnerships with PAHO’s Strategic Fund can also increase access to medicines and other strategic control measures, such as dog collars and mosquito nets, and assist with capacity building in competencies such as demand forecasting, drug registration, and pharmacovigilance. Exploration of the cost-effectiveness of alternative treatments that may be more acceptable to local populations, such as the orally-administered miltefosine and the use of thermotherapy and intralesional pentavalent antimoniate, is on the agenda in Colombia. Under its new Public Health 10-year Plan and leishmaniasis guidelines, a shift in strategy from a medical approach [focused on acquiring and delivering medicines) to a prevention approach (furthering awareness raising and the involvement of community members in leishmaniasis control activities) is foreseen. The treatment guidelines will be a useful tool not only for identifying actions to be taken at each level of the health system but also for involving providers in its roll-out, assessing training and supervisory requirements, and responding to questions or concerns they may have.

The 2022–2031 Public Health Plan and the renewed dedication of MSPS officials provide a window of opportunity for a concerted health system response through committed leadership, teamwork, intersectoral actions, and partnerships with international organizations that share the same goals. This aligns with a recent WHO position paper that emphasizes a whole-of-government approach to ensure community engagement and the involvement of all parts of society [39], and the renewed focus on primary health care after COVID-19, including a rethink of the role of primary health care in pandemic preparedness and response to communicable disease outbreaks [40]. A recent flagship World Bank report on “Reimagining Primary Health Care after COVID-19” also highlights how the pandemic brought to light the challenge of weak health systems globally and their often disastrous consequences. The report also outlines opportunities for rethinking outdated structural and financing models, and potential financing avenues for their implementation [41].

COVID-19 demonstrated the interdependency between disciplines, including human, animal, plant, and environmental health, as is described in the “One Health” approach, a global strategy that advocates for multisectoral and transdisciplinary perspectives across human, animal, plant, and environmental health, recognizing their interconnectedness to achieving better public health outcomes [42,43]. “One Health” provides a framework for multi-stakeholder collaboration, breaking down barriers across relevant health professions, including the sharing of information, knowledge, and experiences, and developing common approaches and solutions [44]. It can help identify early warning signs for transmissible diseases, such as leishmaniasis, and an earlier, more rapid, and more effective response to potential outbreaks [45]. Opportunities to apply the One Health approach to leishmaniasis control have been signaled by PAHO/WHO [43] to strengthen health systems in the countries of the Americas, reduce risks from zoonotic epidemics and pandemics, control and eliminate endemic zoonotic, neglected tropical, and vector-borne diseases, strengthen the assessment, management and communication of food safety risks, curb the pandemic of antimicrobial resistance [43], and integrate environmental concerns into One Health. With regard to VL in particular, a One Health approach encourages joint interventions and monitoring, involving various stakeholders, such as dog owners, private and public veterinarians, and public health practitioners [44]. It also supports surveillance and coordination between veterinary and public health databanks and the use of regional information systems, such as PAHO *SisLeish* in Latin America and the Caribbean that collects and collates epidemiological and operational indicators to inform risk stratification [10]. Furthermore, given its close links to strengthening One Health, NTD control efforts, such as those focused on leishmaniasis, should be better integrated into high-profile pandemic preparedness and response initiatives, particularly in the negotiations for a pandemic accord and amendments to the International Health Regulations (2005), in order to ensure coherent investments in preventing animal to human zoonoses [46].

Regional stakeholders such as PAHO/WHO can be key in supporting elimination initiatives. For example, as pointed out by Colombian officials, assistance is needed to evaluate international purchase opportunities for the most appropriate inputs. Furthermore, while there is a plethora of promotional material on elimination targets and goals, detailed regional or global guidelines for helping programs evaluate their progress in fulfilling the conditions for elimination—such as structural requirements, key process indicators, intermediate results, and impact criteria—are lacking. Such guidelines are needed to help programs plan their logistical and capacity-building needs, adjust accordingly, and evaluate their progress vis-a-vis other countries. In this respect, the lack of dedicated human resources for leishmaniasis is a pressing concern, and ways to address this will need to be found. The possibility of generating teams of national experts from different departments and backgrounds, while aligning them to address common goals, was suggested by the authorities as a possible model to help bridge this gap. This model has been successfully deployed at the international level by organizations such as PAHO/WHO, and mirrors recent recommendations from the World Bank report, mentioned above, that emphasizes collaborative and integrated team-based approaches, the revamping of medical education accordingly, and incentives to encourage health teams to serve in neglected and impoverished communities [41].

Ultimately, the reduction of outbreaks of leishmaniasis will necessitate addressing the social determinants of health, including better sanitary and environmental conditions, nutrition, and investments in the provision of more equitable opportunities for marginalized populations in endemic areas. Even in Nepal, new geographical areas of VL transmission have been recently identified, which seem to be at least partially due to social and economic instability, population movements, and poor housing conditions [47]. The priority given to human rights, diversity, intersectionality, human and social development, and social protection in Colombia’s new 10-year Plan is thus a pivotal opportunity to enhance measures to reduce socioeconomic inequities and the adverse effects of leishmaniasis and other NTDs among poor and marginalized populations. Collaboration with international organizations, such as PAHO’s Strategic Fund, presents important opportunities to source quality-assured and more affordable medicines and supplies to strengthen leishmaniasis prevention and treatment within its essential public health services.

## Supporting information

S1 Table Details of the literature sources reviewed. (DOCX)
