# Supplementary material for: Ensuring access to essential health products: Lessons from Colombia’s leishmaniasis control and elimination initiative
Source: PLoS Negl Trop Dis. 2023 Dec 1;17(12):e0011752. doi: 10.1371/journal.pntd.0011752 (PMC10691678; doi:10.1371/journal.pntd.0011752)
Supplement: S1 Table — (DOCX) [file pntd.0011752.s001.docx]

**Supplemental Table S1:** Details of the literature sources reviewed

| Theme and Date Range | Number searched | Number  selected/  reviewed | Key authoritative documents  (Reference Number) |
| --- | --- | --- | --- |
| Global Policies – *2000 to present*  (U.N., PAHO/WHO, World Bank, CDC, etc.) | 41 | 14 | 1 |
| NTD Policies – *2000 to present* | 8 | 8 | 2,3,4 |
| Leishmaniasis Action Plans – General literature – *2000 to present* | 4 | 4 | 5.6 |
| Leishmaniasis Control – *No cut-off date*  General literature (Epidemiology, Prevention, Treatments, Environment, Community, Population) | 139 | 104 | 7 |
| Colombia Official Documents – *2000 to present* | 13 | 3 | 8 |
| Colombia Leishmaniasis Control – *No cut-off date*  (Epidemiology, Prevention, Treatments, Environment, Community, Population) | 24 | 24 | 9 |
| COVID-19 and Health Systems - *2020 to present* | 6 | 6 | 10 |
| One Health – *No cut-off date* | 15 | 12 | 11 |
| TOTAL | 250 | 175 |  |

References

1. Universal Health Coverage Partnership. Tokyo Declaration on Universal Health Coverage: All Together to Accelerate Progress towards UHC. 2017. [cited 2022 October 6] Available from: https://extranet.who.int/uhcpartnership/news/tokyo-declaration-universal-health-coverage-all-together-accelerate-progress-towards-uhc

2. World Health Organization Ending the neglect to attain the Sustainable Development Goals: A road map for neglected tropical diseases 2021–2030. 2020. Geneva. [cited 6 Oct 2022]. Available: https://www.who.int/publications/i/item/9789240010352

3. PAHO/WHO, 2019. Iniciativa de la OPS para la eliminación de enfermedades: Política para aplicar un enfoque integrado y sostenible de las enfermedades transmisibles en la Región de las Américas. 2019. Washington, D.C. [cited 2022 October 6] Available from: https://www.paho.org/sites/default/files/2020-01/2019-cde-dc57-elimin-init-framework-es.pdf

4. Pan American Health Organization. Leishmaniasis. Epidemiological Report for the Americas. 2022. No.11. [cited 2023 Sept 5] Available from: <https://iris.paho.org/handle/10665.2/56831>

5, Pan American Health Organization. Plan of Action to Strengthen the Surveillance and Control of Leishmaniasis in the Americas 2017-2022. 2017. [cited 2023 Sept 5] Available from: https://iris.paho.org/handle/10665.2/34147

6. Pan American Health Organization. Interactive Atlas of Leishmaniasis in the Americas: Clinical Aspects and Differential Diagnosis. Washington, D.C. 2020 [cited 2022 Jan 13] Available from: https://iris.paho.org/handle/10665.2/53166

7. Georgiadou SP, Makaritsis KP, Dalekos GN. Leishmaniasis revisited: Current aspects on epidemiology, diagnosis and treatment. J Transl Int Med. 2015 Apr-Jun;3(2):43-50. doi: 10.1515/jtim-2015-0002. Epub 2015 Jun 30. PMID: 27847886; PMCID: PMC4936444

8 Ministerio de Salud y Protección Social. 2022. Plan Decenal de Salud Pública, PDSP, 2022-2031. [cited 2023 Sept 5] Available from: https://www.minsalud.gov.co/sites/rid/Lists/BibliotecaDigital/RIDE/VS/ED/PSP/documento-plan-decenal-salud-publica-2022-2031.pdf

9 Ministerio de Salud y Protección Social, Subdirección de Enfermedades Transmisibles, Grupo de Enfermedades Endemoepidémicas. Lineamientos para la atención clínica integral de leishmaniasis en Colombia. Versión 4. 2023. [cited 2022 Oct 6] Available from: [https://www.minsalud.gov.co/sites/rid/Lists/BibliotecaDigital/RIDE/VS/PP/PAI/Lineamientos-leishmaniasis.pdf](https://www.minsalud.gov.co/sites/rid/Lists/BibliotecaDigital/RIDE/VS/PP/PAI/Lineamientos-leishmaniasis.pdf%20Accessed%20Sept%206%202023)

10 Barış E, Silverman R, Wang H; Zhao F, Pate MA.  Walking the Talk: Reimagining Primary Health Care after COVID-19. 2021. World Bank, Washington, DC. http://hdl.handle.net/10986/35842 License: [CC BY 3.0 IGO](http://creativecommons.org/licenses/by/3.0/igo)

11 Feri M, Lloyd-Evans M. The contribution of veterinary public health to the management of the COVID-19 pandemic from a One Health perspective. One Health. 2021 Jun;12:100230, doi: 10.1016/j.onehlt.2021.100230
